# Evaluating Clinical Outcomes in Patients Being Treated Exclusively via Telepsychiatry: Retrospective Data Analysis

**DOI:** 10.2196/53293

**Published:** 2023-12-08

**Authors:** Cheryl Person, Nicola O'Connor, Lucy Koehler, Kartik Venkatachalam, Georgia Gaveras

**Affiliations:** 1 Talkiatry New York, NY United States; 2 McGovern Medical School University of Texas- Health Science Center Houston, TX United States

**Keywords:** telepsychiatry, PHQ-8, GAD-7, clinical outcomes, rural, commercial insurance, telehealth, depression, anxiety, telemental health, psychiatry, Generalized Anxiety Disorder-7, Patient Health Questionnaire-8

## Abstract

**Background:**

Depression and anxiety are highly prevalent conditions in the United States. Despite the availability of suitable therapeutic options, limited access to high-quality psychiatrists represents a major barrier to treatment. Although telepsychiatry has the potential to improve access to psychiatrists, treatment efficacy in the telepsychiatry model remains unclear.

**Objective:**

Our primary objective was to determine whether there was a clinically meaningful change in 1 of 2 validated outcome measures of depression and anxiety—the Patient Health Questionnaire–8 (PHQ-8) or the Generalized Anxiety Disorder–7 (GAD-7)—after receiving at least 8 weeks of treatment in an outpatient telepsychiatry setting.

**Methods:**

We included treatment-seeking patients enrolled in a large outpatient telepsychiatry service that accepts commercial insurance. All analyzed patients completed the GAD-7 and PHQ-8 prior to their first appointment and at least once after 8 weeks of treatment. Treatments included comprehensive diagnostic evaluation, supportive psychotherapy, and medication management.

**Results:**

In total, 1826 treatment-seeking patients were evaluated for clinically meaningful changes in GAD-7 and PHQ-8 scores during treatment. Mean treatment duration was 103 (SD 34) days. At baseline, 58.8% (1074/1826) and 60.1% (1097/1826) of patients exhibited at least moderate anxiety and depression, respectively. In response to treatment, mean change for GAD-7 was –6.71 (95% CI –7.03 to –6.40) and for PHQ-8 was –6.85 (95% CI –7.18 to –6.52). Patients with at least moderate symptoms at baseline showed a 45.7% reduction in GAD-7 scores and a 43.1% reduction in PHQ-8 scores. Effect sizes for GAD-7 and PHQ-8, as measured by Cohen *d* for paired samples, were *d*=1.30 (*P*<.001) and *d*=1.23 (*P*<.001), respectively. Changes in GAD-7 and PHQ-8 scores correlated with the type of insurance held by the patients. Greatest reductions in scores were observed among patients with commercial insurance (45% and 43.9% reductions in GAD-7 and PHQ-8 scores, respectively). Although patients with Medicare did exhibit statistically significant reductions in GAD-7 and PHQ-8 scores from baseline (*P*<.001), these improvements were attenuated compared to those in patients with commercial insurance (29.2% and 27.6% reduction in GAD-7 and PHQ-8 scores, respectively). Pairwise comparison tests revealed significant differences in treatment responses in patients with Medicare versus commercial insurance (*P*<.001). Responses were independent of patient geographic classification (urban vs rural; *P*=.48 for GAD-7 and *P*=.07 for PHQ-8). The finding that treatment efficacy was comparable among rural and urban patients indicated that telepsychiatry is a promising approach to overcome treatment disparities that stem from geographical constraints.

**Conclusions:**

In this large retrospective data analysis of treatment-seeking patients using a telepsychiatry platform, we found robust and clinically significant improvement in depression and anxiety symptoms during treatment. The results provide further evidence that telepsychiatry is highly effective and has the potential to improve access to psychiatric care.

## Introduction

### Background

Depression and anxiety are the 2 most common mental health conditions in the United States and worldwide, ranking second and eighth, respectively, in the list of causes for disability [1]. The chronicity and prevalence of these illnesses have an enormous economic impact and have worsened the quality of life in countries across the globe. A recent health economics review found that depression alone accounts for roughly 16% of the loss of work-related productivity [2]. Those who have depression and anxiety have worse health outcomes if left untreated [3-5].

Although effective treatment is available for both these chronic conditions, access to these treatments can be a challenge, which has led to significant gaps in treatment [6-8]. Difficulties in access to treatment can stem from physical distances from a treatment site, difficulties with arranging for time away from home or work to attend clinical appointments, prohibitive treatment costs, and the paucity of specialist care [9,10]. Remarkably, 27% of the counties in the United States are characterized by the complete absence of psychiatrists [11]. Rural residents are noteworthy for having limited access to in-person psychiatric care [9,11]. Even more troubling, it appears that issues of access occur independently of the patients’ health insurance status. A recent survey found that 31% of US adults reported the inability to access mental health services despite being insured [12]. Further, the medical specialty least likely to participate in health insurance plans is psychiatry [13]. Consequently, the total cost for outpatient psychiatric care is often not mitigated by health insurance. Not only is outpatient psychiatric care without insurance often cost prohibitive, the potential paucity of in-network psychiatrists may further delay access to treatment. These delays in access to mental health treatment are burdensome to patients and frequently worsen other associated medical conditions [14,15].

The aforementioned obstacles are especially disconcerting given the efficacy of treatments for both depression and anxiety. Antidepressants and psychotherapy have long been recognized as being beneficial for treating these conditions [16,17]. Given that modern technology permits the delivery of evidence-based treatments remotely and that psychiatry as a practice does not require the presence of patients and psychiatrists in the same physical space, telepsychiatry is a valuable tool for overcoming these persistent barriers. Indeed, the recent easing of regulatory obstacles to the implementation of telepsychiatry platforms has led to a vast expansion in the ease of access to mental health services. Encouragingly, a growing body of literature indicates that patients receiving telehealth treatments exhibit improvements that are comparable to those seen in patients receiving in-person care [18-20]. Others have demonstrated that a combination of flexibly implemented psychotherapy and medication management is effective for the management of anxiety disorders [21]. Presently, the potential efficacy of telepsychiatry services for patients who elect a fully virtual telepsychiatry service remains unknown.

In this study, we examined the outcomes of a large private outpatient telepsychiatry practice. Talkiatry is a fully virtual, nationwide outpatient private psychiatry practice that accepts commercial health insurance. This practice employs over 300 psychiatrists and more than 50 therapists across the United States. Although virtual, Talkiatry uses the traditional psychiatric model of care—an initial comprehensive evaluation, followed by the development of a treatment plan that revolves around patient preferences. This type of patient-centric approach comprised of partnerships between physicians and patients that are built on the foundation of effective communication and health promotion has been found to improve medical outcomes and forms the basis of effective outpatient psychiatric care [22].

The concept of therapeutic alliance—the collaborative relationship between the patient and physician—is another core element of psychiatric care that has been described as one of the most valuable aspects of treatment effectiveness with strong positive impact on outcomes in psychotherapy [23-25]. In fact, a patient’s adherence to treatment is often motivated by a strong therapeutic alliance [26]. Further, recent studies have suggested that therapeutic alliance is the mediator of change in psychotherapy studies across a broad range of modalities and conditions [27]. The efficacy of therapeutic alliance depends on the ability of the physician to convey empathy and friendliness, to understand a patient’s unique set of motivations and interests, and to implement an individualized treatment that is personalized to that patient’s preferences [28]. Not surprisingly, it takes time to build a therapeutic alliance. Therefore, the establishment of therapeutic alliance is difficult in the common and increasingly dominant 15-minute medication checks that characterize current psychiatric practice [29,30]. Here, we examined whether the Talkiatry telepsychiatry model, which has time set aside specifically for the establishment of a strong therapeutic alliance, led to improved clinical outcomes for patients with anxiety or depression.

### Objectives

The primary objective of this study was to determine whether there was a clinically meaningful change in 1 of 2 validated outcome measures—the Patient Health Questionnaire–8 (PHQ-8) or the Generalized Anxiety Disorder–7 (GAD-7)—after receiving treatment in the outpatient telepsychiatry practice for a minimum of 8 weeks. This retrospective data analysis reflects real-world treatment conditions where the combination of therapy and medication management remains the primary modality of treatment. Our secondary objectives were to determine if patient demographic characteristics (sex, urban or rural, initial GAD-7 or PHQ-8 severity score, age, and insurance status) correlated with statistically significant changes in treatment outcomes.

## Methods

### Study Cohort and Treatment Strategy

This study’s population was composed of treatment-seeking individuals who had enrolled in an outpatient telepsychiatry practice. Patients sought enrollment in services through several commercially available booking platforms or the company website, and they were required to complete of a brief web-based questionnaire. Enrollment was restricted to individuals who did not present with primary psychotic disorder, primary substance use disorder, recent psychiatric hospitalization, current suicidal ideation, and insurance types not accepted by the practice. Once screening was completed, patients were provided several psychiatrists’ profiles to help them choose the provider that met their unique preferences (gender, condition specialty, and availability outside of business hours). From that curated group, each patient selected a psychiatrist and scheduled a new patient evaluation. All patients were requested to complete the GAD-7 and PHQ-8 measures up to 7 days prior to their first appointment and approximately every month thereafter. This study’s sample was composed of patients who (1) were 18 years of age or older, (2) had two sequential appointments with a treating psychiatrist at least 8 weeks apart, and (3) had completed either one or both GAD-7 and PHQ-8 measures twice (prior to each appointment). This study’s timeframe was the completion of initial visit between February 2023 and June 2023, with a subsequent visit completed at least 8 weeks later but before September 2023. If a patient completed multiple measures, the last completed measure was selected for analysis.

Therapy and medication management were provided in an audiovisual format that used a commercially available telehealth platform. Psychiatrists conducted comprehensive psychiatric evaluations and, in collaboration with the patient, developed appropriate treatment plans. Ongoing treatments, which included supportive psychotherapy, developing therapeutic alliance, and medication management, were embedded within the appointments. On average, psychiatrists spent 26 minutes of each patient visit on therapy. Each patient included in this study was seen by a psychiatrist at least twice.

### Ethical Considerations

All data in this study were routinely collected as part of standard clinical practice. This study was reviewed and granted exempt status by the institutional review board of Advarra (Pro00073660). A full waiver of Health Insurance Portability and Accountability Act (HIPAA) authorization was granted under expedited review process. All data were deidentified by the data privacy team prior to all subsequent analyses.

### Measures

The PHQ-8 is an 8-item self-reporting questionnaire that quantifies the symptoms of depression and measures progress in patient outcomes over time [31]. The scale performs similarly to the PHQ-9 and is routinely used in telehealth research and clinical practice settings [32]. For the PHQ-8, the standard cutoff scores were used to categorize patients into minimal (0-4), mild (5-9), moderate (10-14), moderately severe (15-19), and severe (20-24) depression. The GAD-7 is a 7-item self-reporting questionnaire that quantifies symptoms of generalized anxiety disorder and tracks progress of patient outcomes over time [33]. For the GAD-7, the standard cutoff scores were used to categorize patients into minimal (0-4), mild (5-9), moderate (10-14), and severe (15-21) anxiety. Initial PHQ-8 and GAD-7 scores were collected within 7 days of the first appointment and at approximately 1-month intervals during treatment. Demographic variables that were analyzed included age, sex, type of health insurance (commercial insurance, Medicare, or self-pay), and urban or rural classification. Self-pay was assigned to patients who were initially insured but changed or lost insurance during the course of treatment.

### Statistical Analysis

Primary outcomes of interest in this study were anxiety and depressive symptoms measured by the GAD-7 and PHQ-8, respectively, assessed at the last visit. Changes in GAD-7 and PHQ-8 scores were calculated by subtracting the last scores from baseline scores for each patient. In order to examine clinically relevant samples, only patients with at least mild symptoms at baseline (ie, GAD-7 score≥5 or PHQ-8 score≥5) were included in the analyses. We use paired sample Wilcoxon tests to examine whether statistically significant changes in GAD-7 and PHQ-8 scores were observed over time (from baseline to last visit) among all patients. Effect sizes for paired 2-tailed *t* tests were also calculated using the SD of the differences. These analyses were repeated with subsamples including patients with at least moderate baseline symptoms (ie, baseline GAD-7 score≥10 and baseline PHQ-8 score≥10).

We examined whether changes in anxiety and depressive symptom scores varied by sex, age group (18-24 years, 25-64 years, and 65+ years), baseline symptoms-severity category, insurance type (ie, commercial insurance, Medicare, and self-pay), and geographical classification (ie, urban vs rural) using Wilcoxon, Kruskal-Wallis, or chi-square tests. Binary outcomes indicating patients that showed clinically significant symptom improvement were 1 for patients with 50% or greater symptoms improvement and 0 for patients not at goal. For these outcomes, we chose to include only those patients with at least moderate baseline symptoms (ie, baseline GAD-7 score≥10 and PHQ-8 score≥10).

We determined the fraction of patients with >50% symptom improvement, including those that exhibited remission (final score<5). Using the chi-square test, we asked whether the fraction of patients with symptom improvement varied as a function of the category of baseline symptom severity. In these analyses, we included patients with at least moderate baseline symptoms because GAD-7 and PHQ-8 scores ≥10 represent a high probability of clinically significant symptoms [31]. Statistical analyses were performed using R (R Foundation for Statistical Computing) and Prism (version 10.0.02; GraphPad).

## Results

### Overview

The demographic and baseline characteristics of the patients are presented in Table 1. During this study’s timeframe, which was between February 2023 and September 2023, a total of 6465 patients enrolled in outpatient care and had available baseline measures. Of those, 1826 participants, representing 28.2% of the initial sample, completed a second measure and were included in the analysis. A total of 1826 participants had data available for analyses and had a mean age of 39.4 (SD 13.5; range 18-89) years. Of those who reported sex (n=1479, 81%; n=347, 19% did not report sex), 1084 (73.3%) were female. The mean number of days between baseline and last visit was 103 (SD 34) days. Of those 1826 patients, 1602 (87.7%) reported at least mild anxiety symptoms (GAD-7 score≥5), while 1572 (86.1%) reported at least mild depressive symptoms (PHQ-8 score≥5) at baseline. Further, 58.8% (1074/1826) of the sample reported at least moderate anxiety symptoms and 60.1% (1097/1826) of the sample reported at least moderate depressive symptoms. Of the original sample, 4639 (71.8%) out of 6465 patients did not complete the second measure. Compared to patients who were noncompleters, those who completed repeated measures were older, more likely to be insured with Medicare, and more likely to be classified as rural. Importantly, they did not differ on baseline severity for either depressive or anxiety symptoms (Table 1).

**Table 1:** Demographic and baseline characteristics.

Baseline variables			Patients with repeat measures (n=1826), n (%)		Patients with 1 measure (n=4639, n (%)		*P* value^a^	
**Sex**								.06
	Female	1084 (59.36)		2620 (56.48)				
	Male	395 (21.63)		1086 (23.41)				
	Undisclosed	347 (19)		933 (20.11)				
Age (y), mean (SD)			39.38 (13.53)		33.32 (10.05)		<.001	
**Insurance type**								<.001
	Commercial insurance	1618 (88.61)		4409 (95.04)				
	Medicare	138 (7.56)		84 (1.81)				
	Self-pay	70 (3.83)		146 (3.15)				
**Baseline GAD-7^b^**								.90
	Minimal anxiety	224 (12.27)		544 (11.73)				
	Mild anxiety	528 (28.92)		1332 (28.71)				
	Moderate anxiety	541 (29.63)		1376 (29.66)				
	Severe anxiety	533 (29.19)		1387 (29.9)				
**Baseline PHQ-8^c^**								.49
	Minimal depression	254 (13.91)		567 (12.22)				
	Mild depression	475 (26.01)		1260 (27.16)				
	Moderate depression	530 (29.03)		1383 (29.81)				
	Moderately severe depression	394 (31.58)		1032 (22.25)				
	Severe depression	173 (9.47)		397 (8.56)				
**Geographic classification**								.01
	Urban	1710 (93.65)		4420 (95.28)				
	Rural	116 (6.35)		218 (4.7)				
	Number of days between visits	102.63 (34.15)		N/A^d^				

^a^*P* values obtained from chi-square tests comparing the group that completed repeated measurements and the group that completed 1 measurement.

^b^GAD-7: Generalized Anxiety Disorder–7.

^c^PHQ-8: Patient Health Questionnaire–8.

^d^N/A: not available as second measurement not completed.

### Changes in Anxiety and Depressive Symptoms

#### Overview

Results from paired sample Wilcoxon tests showed that among patients with at least mild baseline symptoms (GAD-7 or PHQ-8 scores≥5), both anxiety (GAD-7 scores) and depressive symptoms (PHQ-8 scores) decreased significantly from the first to last visit. The effect sizes for the GAD-7 and PHQ-8, as measured by Cohen *d* for paired samples, were *d*=1.05 (*P*<.001) and *d*=0.98 (*P*<.001), respectively. These findings indicated large effects (43.7% reductions in GAD-7, 42.7% reductions in PHQ-8). Furthermore, these findings were replicated when the analyses were conducted with the subsample of patients that exhibited at least moderate baseline symptoms (GAD-7 or PHQ-8 scores≥10). We observed larger effect sizes of *d*=1.30 (*P*<.001) for GAD-7 (45.7% reductions) and *d*=1.23 (*P*<.001) for PHQ-8 (45.1% reductions) in the subsample. These data indicated that greater reductions in anxiety and depressive symptoms occurred among patients with more severe symptoms at baseline (Table 2).

**Table 2:** Changes in anxiety and depression symptoms in patients with at least mild baseline symptoms. Patients with at least mild baseline symptoms (baseline GAD-7^a^ or PHQ-8^b^ scores≥5) are included in the analyses.

Measure and sample		Responses, n	Baseline score, mean (SD)	Last score, mean (SD)	Changes from baseline (%)	Mean difference (95% CI)	Effect size (Cohen *d*)	Baseline score, median	Last score, median	Paired sample Wilcoxon test	*P* value
**GAD-7**											
	Baseline GAD-7 score≥5	1602	12.20 (4.55)	6.87 (4.82)	43.69	–5.34 (–5.59 to –5.09)	1.05	12	6	V=1099198	<.001
	Baseline GAD-7 score≥10	1074	14.72 (3.27)	8.00 (5.00)	45.65	–6.71 (–7.03 to –6.40)	1.30	14	7	V=1039814	<.001
**PHQ-8**											
	Baseline PHQ-8 score≥5	1572	12.78 (4.90)	7.33 (5.47)	42.64	–5.45 (–5.73 to –5.18)	0.98	12	6	V=519504	<.001
	Baseline PHQ-8 score≥10	1097	15.22 (3.74)	8.37 (5.69)	45.01	–6.85 (–7.18 to –6.52)	1.23	15	8	V=482402	<.001

^a^GAD-7: Generalized Anxiety Disorder–7.

^b^PHQ-8: Patient Health Questionnaire–8.

#### Sex

Kruskal-Wallis tests showed that the overall fraction of patients with 50% or greater reductions in either GAD-7 or PHQ-8 scores did not differ by sex (GAD-7: *P*=.33; PHQ-8: *P*=.10).

#### Baseline Symptom Severity

Using Kruskal-Wallis tests to examine whether change in GAD-7 and PHQ-8 scores differed across baseline symptom severity category, we found greater reductions among those with more severe baseline symptoms. The greatest reductions were among patients with severe baseline symptoms (GAD-7 score≥15 and PHQ-8 score≥20). The smallest reductions were among patients with mild baseline symptoms (GAD-7 or PHQ-8 scores=5-9; Table 3). Pairwise comparisons using Wilcoxon test with continuity correction revealed that significant differences in change in GAD-7 and PHQ-8 scores were observed between all comparison pairs (all *P*<.01). Therefore, groups with more severe baseline symptoms showed significantly greater reductions in anxiety and depressive symptoms compared to any of the groups with lower baseline severity.

**Table 3:** Mean difference in anxiety and depression symptoms by baseline severity category. Patients with at least mild baseline symptoms (baseline GAD-7^a^ or PHQ-8^b^ scores≥5) are included in the analyses.

Measure and baseline severity			Responses, n (%)		Baseline score, mean (SD)		Last score, mean (SD)		Changes from baseline (%)		Mean difference (95% CI)		Baseline score, median		Last score median		Kruskal-Wallis (*df*)			*P* value	
**GAD-7 (n=1602)**																		336.85 (2)			<.001
	Mild anxiety (5-9)	528 (32.96)		7.09 (1.39)		4.55 (3.42)		35.83		–2.54 (–2.84 to –0.24)		7		4							
	Moderate anxiety (10-14)	541 (33.77)		11.91 (1.37)		6.79 (4.08)		42.99		–5.12 (–5.47 to –4.77)		12		6							
	Severe anxiety (≥15)	533 (33.27)		17.57 (1.88)		9.24 (5.51)		47.41		–8.33 (–0.81 to –7.86)		17		8							

**PHQ-8 (n=1572)**																		304.6 (3)			<.001
	Mild depression (5-9)	475 (30.22)		7.15 (1.34)		4.93 (4.01)		31.05		–2.22 (–2.59 to –1.86)		7		4							
	Moderate depression (10-14)	530 (33.72)		11.97 (1.40)		6.68 (4.61)		44.19		–5.29 (–5.80 to –4.90)		12		6							
	Moderately severe depression (15-19)	394 (25.06)		16.91 (1.39)		9.08 (5.94)		46.30		–8.83 (–8.41 to –7.24)		17		8							
	Severe depression (≥20)	173 (11.01)		21.36 (1.31)		11.95 (6.13)		44.05		–9.41 (–10.31 to –8.51)		21		12							

^a^GAD-7: Generalized Anxiety Disorder–7.

^b^PHQ-8: Patient Health Questionnaire–8.

#### Insurance Type

Insurance types (ie, commercial insurance, Medicare, and self-pay) also predicted change scores of both anxiety symptoms (GAD-7) and depressive symptoms (PHQ-8). Greatest reductions were observed among those with commercial insurance (a 45% reduction for GAD-7 scores and a 43.9% reduction in PHQ-8 scores), followed by those with self-pay (a 40.8% reduction for GAD-7 scores and a 40.1% reduction in PHQ-8 scores) and Medicare (a 29.2% reduction for GAD-7 scores and a 27.6% reduction in PHQ-8 scores). Pairwise comparison tests revealed that significant differences in change scores were found between Medicare and commercial insurance (*P*<.001) and Medicare and self-pay (*P*<.01), but not commercial insurance and self-pay (*P*=.74; Table 4).

**Table 4:** Changes in anxiety and depression symptoms by insurance type^a^.

Measure and insurance type			Responses, n (%)		Baseline score, mean (SD)		Last score, mean (SD)		Changes from baseline (%)	Mean difference (95% CI)		Baseline score, median		Last score, median		Kruskal-Wallis (*df*)		*P* value	
**GAD-7^b^ (n=1602)**																	14.04 (2)		<.001
	Commercial insurance	1424 (88.9)		12.21 (4.54)		6.72 (4.72)		44.96		–5.50 (–5.76 to –5.23)	12		6						
	Medicare	115 (7.2)		11.61 (4.36)		8.22 (5.41)		29.20		–3.39 (–4.31 to –2.47)	11		7						
	Self-pay	63 (3.9)		13.11 (5.02)		7.76 (5.44)		40.81		–5.35 (–6.54 to –4.16)	14		6						
**PHQ-8^c^ (n=1572)**																	20.46 (2)		<.001
	Commercial insurance	1405 (89.3)		12.76 (4.92)		7.16 (5.40)		43.89		–5.60 (–5.89 to –5.31)	12		6						
	Medicare	107 (6.8)		12.62 (4.930)		9.14 (5.70)		27.58		–3.48 (–4.55 to –2.40)	12		8						
	Self-pay	60 (3.8)		13.60 (4.43)		8.15 (6.13)		40.07		–5.45 (–6.77 to –4.13)	13		6.5						

^a^Patients with at least mild baseline symptoms (baseline GAD-7 or PHQ-8 scores≥5) are included in the analyses.

^b^GAD-7: Generalized Anxiety Disorder–7.

^c^PHQ-8: Patient Health Questionnaire–8.

#### Age Group

In the unadjusted model, age groups predicted differences in the fraction of patients with 50% or greater reductions in both GAD-7 (*χ*^2^_2_=11.44, n=1826, *P*=.003) and PHQ-8 scores (χ^2^_2_=9.57, n=1826, *P*=.008).

#### Geographic Classification (Urban vs Rural)

No differences in change in either GAD-7 or PHQ-8 scores were found across geographic classification (ie, urban vs rural), indicating that similar reductions in symptoms were observed across patients’ geographic location (Table 5).

**Table 5:** Changes in anxiety and depression symptoms by geographical classification^a^.

Measure and geographic classification		Responses, n (%)	Baseline score, mean (SD)	Last score, mean (SD)	Changes from baseline (%)	Mean difference (95% CI)	Baseline score, median	Last score, median	Wilcoxon rank sum test (*df*)		*P* value	
**GAD-7^b^ (n=1602)**										W=80435 (1)		.48
	Urban	1499 (93.58)	12.19 (4.57)	7.45 (4.88)	38.88	–5.36 (–5.62 to –5.11)	12	6				
	Rural	103 (6.43)	12.41 (4.26)	6.83 (4.81)	44.96	–4.96 (–5.92 to –4.00)	12	6				
**PHQ-8^c^ (n=1572)**										W=70020 (1)		.07
	Urban	1465 (93.19)	12.74 (4.90)	7.35 (5.47)	42.31	–5.29 (–5.67 to –5.10)	12	6				
	Rural	107 (6.80)	13.47 (4.95)	7.12 (5.62)	47.14	–6.35 (–7.39 to –5.30)	13	5				

^a^Patients with at least mild baseline symptoms (baseline GAD-7 or PHQ-8 scores≥5) are included in the analyses.

^b^GAD-7: Generalized Anxiety Disorder–7.

^c^PHQ-8: Patient Health Questionnaire–8.

### Clinically Significant Symptom Improvement (50% or Greater Reductions in GAD-7 and PHQ-8 Scores)

#### Overview

Of the 1074 patients who reported at least moderate anxiety symptoms at baseline (GAD-7 score≥10), 574 (53.45%) showed clinically significant improvement of anxiety symptoms (50% or greater reductions in GAD-7 scores). This included 31% (n=86) of the patients in the moderate category and 21% (n=62) of patients in the severe category that achieved remission. Among the 1097 patients who reported at least moderate depressive symptoms at baseline (PHQ-8 scores≥10), 562 (51.23%) patients showed clinically significant symptoms improvement for depressive symptoms (50% or greater reductions in PHQ-8 scores). This included 37% (n=101), 27% (n=56), and 13% (n=10) of patients in the moderate, moderately severe, and severe categories who achieved remission. Of the 1074 patients who reported at least moderate anxiety symptoms at baseline, 724 (67.41%) showed only minimal or mild symptoms at last visit. Of the 1097 patients who reported moderate or greater depressive symptoms at baseline, 685 (62.44%) reported only mild or minimal symptoms at last visit (Table 6).

**Table 6:** Percentage of patients with at least moderate baseline symptoms who achieved >50% improvement or had final severity of minimal or mild symptoms^a^.

Measure	Responses, n	Patients with 50% or greater symptom improvement, n (%)	Patients with final score of minimal or mild symptoms^b^, n (%)
GAD-7^c^	1074	574 (53.45)	724 (67.41)
PHQ-8^d^	1097	562 (51.23)	685 (62.44)

^a^Patients with at least moderate baseline symptoms (baseline GAD-7 or PHQ-8 scores≥10) are included in the analyses.

^b^Minimal or mild symptoms: PHQ-8 or GAD-7<10.

^c^GAD-7: Generalized Anxiety Disorder–7.

^d^PHQ-8: Patient Health Questionnaire–8.

#### Baseline Symptom Severity

Among those with at least moderate baseline symptoms, the rates of patients with 50% or greater reductions in either anxiety symptoms (GAD-7) or depressive symptoms (PHQ-8) did not differ across baseline symptom severity category (*P*=.17 and *P*=.34 respectively). This indicates that fractions of patients who showed clinically significant symptom improvement were similar across patients with moderate and severe baseline symptoms (Table 7).

**Table 7:** Percentage of patients with >50% symptom improvement by baseline severity category. Patients with at least moderate baseline symptoms (baseline GAD-7^a^ or PHQ-8^b^≥10) are included in the analyses.

Measure and baseline severity		Responses (GAD-7: n=1074; PHQ-8: n=1097), n (%)	Patients with 50% or greater symptom improvement, n (%)^c^	*χ*^2^ (*df*)		*P* value
**GAD-7**					1.8 (1, n=1074)	.17
	Moderate anxiety (10-14)	541 (50.37)	278 (51.38)			
	Severe anxiety (≥15)	533 (49.63)	296 (55.53)			
**PHQ-8**					2.1 (2, n=1097)	.34
	Moderate depression (10-14)	530 (48.31)	274 (51.70)			
	Moderately severe depression (15-19)	394 (35.92)	208 (52.79)			
	Severe depression (≥20)	173 (15.77)	80 (46.24)			

^a^GAD-7: Generalized Anxiety Disorder–7.

^b^PHQ-8: Patient Health Questionnaire–8.

^c^The denominators are the values in the “Responses” column.

## Discussion

### Principal Findings

In this retrospective study, we found that telepsychiatry treatment outcomes for depression and anxiety were statistically and clinically significant. Telepsychiatry treatment led to average reductions of 45.7% for anxiety symptoms and 45.1% for depressive symptoms. Patients with baseline anxiety or depression with severity in the moderate to severe ranges exhibited improved outcomes at the last visit—67.41% (724/1074) of patients with anxiety and 62.44% (685/1097) of patients with depression reported minimal or mild symptoms in response to an average of 103 days of treatment. Further, mean difference between initial and final scores were from 42% to 45%, which points to robust clinical responses. Interestingly, the extent of improvement was not a function of baseline severity indicating that the efficacy of treatment was comparable among patients that exhibited either moderate or severe symptoms at the start of treatment.

Our findings are consistent with prior research on efficacy of therapeutic alliance in treatment and provide evidence in support of the notion that therapeutic alliance can be achieved in a telepsychiatry setting. Unique aspects of this psychiatric model include longer visits to allow for the development of effective therapeutic alliance, as well as allowing patients to use their health insurance, which increases access to treatment and reduces the total cost of care. Improving access by reducing cost, reducing delays in seeking treatment, and having confidence in the working relationship with the psychiatrist are important components that improve clinical outcomes in a telepsychiatry platform.

We also found that among individuals using telepsychiatry, rural patients benefitted to the same extent as did urban patients. This finding is promising because it is known that rural patients frequently struggle with access to care due to difficulties with either internet connectivity or geographical constraints [34]. While this study does not address the issue of internet connectivity, our findings argue that in the presence of acceptable internet quality, rural patients were able to access telepsychiatry treatments and showed improvements that were comparable to those seen in their urban counterparts.

Another demographic feature we examined was insurance type. While patients with Medicare insurance in the telepsychiatry practice did exhibit symptom improvements over the course of treatment, the extent of improvement was lower than that observed in patients with commercial insurance or self-pay. This finding is consistent with prior reports that Medicare beneficiaries typically take a longer time to respond to treatment and have more overall disease burden, which impacts improvement [35,36].

### Limitations

This study has some limitations. Using data from a private practice and using telepsychiatry as the exclusive mode of treatment, we did not have access to a control group that received in-person treatment. This precludes a direct comparison to in-person studies. That being said, we have found that our depression and anxiety outcomes are similar to other such studies [18,37]. A second limitation in this study is that our sample had a comparatively small number of Medicare beneficiaries and a small number of patients older than 65 years. Due to this limitation, we were unable to determine which of these variables (age, Medicare status, or both) impacted the mean improvement scores. Further, there were more rural residents who completed both measures, and while rural residents did not differ in treatment outcomes from urban residents, they may have differed from rural residents who only completed 1 measure. Another limitation is that other psychiatric conditions that were excluded, such as primary substance use disorder, primary psychotic disorder, and primary eating disorder, may limit generalizability of the results. As there is considerable comorbidity with other psychiatric illnesses, these treatment outcomes may not be fully representative of all patients who have depression and anxiety. Lastly, although our data are consistent with the establishment of an effective therapeutic alliance, this retrospective analysis lacks quantitative measures of therapeutic alliance. Incorporating measures of therapeutic alliance into routine clinical practice could allow direct evaluation of this relationship in the future.

### Comparison With Prior Work

This study adds to the emerging body of literature that is showing that telepsychiatry can be an effective treatment modality for many mental health conditions [37,38].

### Conclusions

In this large real-world retrospective data analysis of patients using a telepsychiatry platform, we found robust and clinically significant improvement in their depression and anxiety symptoms during the course of treatment. For patients with moderate to severe symptoms of either anxiety or depression, the improvements are similar and confirm that patients with severe illness can be effectively treated in a telepsychiatry practice. This adds to the growing body of medical literature that telepsychiatry can be effective.

## Abbreviations

GAD-7: Generalized Anxiety Disorder–7
HIPAA: Health Insurance Portability and Accountability Act
PHQ-8: Patient Health Questionnaire–8

## Data Availability

The data are available from the corresponding author upon reasonable request. Only deidentified data will be made available.
